# Real-Life Efficacy of Bevacizumab Treatment for Macular Edema Secondary to Central Retinal Vein Occlusion according to *Pro Re Nata* or Treat-and-Extend Regimen in Eyes with or without Epiretinal Membrane

**DOI:** 10.1155/2022/6288582

**Published:** 2022-10-03

**Authors:** Moustafa Hamam, Neil Lagali, Elie Abdulnour, Helen Setterud, Björn Johansson, Pierfrancesco Mirabelli

**Affiliations:** Department of Ophthalmology and Department of Biomedical and Clinical Sciences, Linköping University, Linköping 581 85, Sweden

## Abstract

**Purpose:**

To present real-life data of patients with macular edema (ME) secondary to central retinal vein occlusion (CRVO) treated with bevacizumab (BVZ); determine the possible influence of epiretinal membrane (ERM) on treatment efficacy; and compare treatment outcomes in a treat-and-extend regimen (TER) versus *pro re nata* (PRN).

**Methods:**

We carried out a retrospective analysis of 58 eyes (56 patients) with new-onset CRVO treated only with intravitreal bevacizumab according to TER or PRN. Outcome measures were best-corrected visual acuity (BCVA) and central retinal thickness (CRT) at baseline and 12 months after the first treatment, number of visits and injections, and presence of ERM confirmed by optical coherence tomography in the first 6 months.

**Results:**

At 12 months, the mean number of injections was 6.3 across all eyes, with significantly more injections given in TER (*p* < 0.001). Mean CRT improved from 627 *μ*m to 359 *μ*m (*p* < 0.001) in all eyes, with improvement noted in TER (*p* < 0.001), PRN (*p* < 0.001), ERM (*p*=0.003), and non-ERM (*p* < 0.001) subgroups. The mean BCVA gain was +13.6 letters, and the mean BCVA improved from 0.81 to 0.54 LogMAR (*p* < 0.001) in all eyes. BCVA improvement from baseline was significant in TER (*p* < 0.001) and non-ERM (*p* < 0.001) but not in PRN (*p*=0.08) or ERM (*p*=0.2) subgroups. Seven eyes, all receiving PRN treatment, developed neovascularization.

**Conclusions:**

Intravitreal bevacizumab according to either PRN or TER resolved edema and stabilized vision in the first 12 months, with TER yielding significant visual improvement and avoiding neovascular complications. ERM had no influence on bevacizumab efficacy in reducing ME in CRVO during 12 months of treatment.

## 1. Introduction

Retinal vein occlusion (RVO) is the second most common retinal vascular disease [1]. Visual loss is most frequent in the central type of RVO (CRVO), with a prevalence of 0.1%–0.4% in individuals over 40 years of age [2, 3]. Age is a primary risk factor for the development of CRVO, with 90% of patients being 50 years or older. Diabetes, glaucoma, arterial hypertension, and hyperlipidemia are also primary risk factors for CRVO. Other risk factors may include hyper-homocysteinemia or other diseases characterized by hyper-coagulation (multiple myeloma, antiphospholipid syndrome, polycythemia, etc.), as well as optic disc edema, infection diseases as, for example, syphilis, retinal or systemic vasculitides, sarcoidosis, optic disc drusen, use of oral contraceptives, and use of diuretics [4].

Retinal vein occlusion is complicated mainly with macular edema and ischemia, the latter causing neovascularization in the retina and iris. When compared with healthy individuals, intraocular Vascular Endothelial Growth Factor (VEGF), which is believed to play a central role in the pathogenesis of neovascularization and macular edema, has been found to be elevated in eyes with CRVO [5]. Macular ischemia and edema as well as sequelae caused by neovascularization are complications of RVO, leading to loss of vision [6, 7].

A comparative investigation of 674 patients with CRVO and hemi-CRVO reported a prevalence of primary open-angle glaucoma of 6.1% in 41 patients, which is significantly higher than in the general population (0.41–2.89%) [8]. In addition, neovascular (NV) glaucoma is the main complication in eyes with the ischemic type of CRVO [9,10]. Pan-retinal photocoagulation (PRP) can regress retinal neovascularization in eyes with CRVO [7, 11–14].

Macular edema occurs mainly due to abnormal vascular permeability, with VEGF playing a central role [15]. In eyes with CRVO, macular edema is the main cause of vision loss, and the use of intravitreal anti-VEGF therapy [11–14, 16] or dexamethasone implants [17, 18] has considerably improved the visual prognosis of patients with CRVO.

Bevacizumab (BVZ), a humanized monoclonal anti-VEGF-A antibody, has been shown in several studies to be effective in the treatment of ME due to RVO (RVO-ME), an off-label indication [19–23]. In a prospective study, SCORE2, monthly treatment with aflibercept or bevacizumab during the first 6 months, gave similar morphological and vision results in RVO-ME patients, showing noninferiority of BVZ versus aflibercept [24]. Another study showed that bevacizumab injections may provide functional and anatomical improvement in eyes with ME secondary to CRVO (CRVO-ME) [25]. Epstein et al., in a prospective study comparing BVZ with sham injections for CRVO-ME, reported a significant gain in visual acuity in the BVZ group [26]. Furthermore, other studies also show good results for BVZ [23, 26–28]. In the County of Östergötland, Sweden, where the present study was conducted, BVZ is the first-line choice for treatment of CRVO-ME.

Anti-VEGF treatment as described in the first Randomized Clinical Trials (RCTs) is usually given as a fixed regimen with monthly injections, followed by a maintenance phase with treatment according to the *pro re nata* (PRN) regimen. The patients are followed-up monthly, and re-injections are given as needed if specific clinical activity criteria are met. The CRUISE study [11] was a prospective RCT that reported 12 months' results of anti-VEGF treatment of CRVO-patients with ranibizumab given with 6 monthly injections followed by PRN. Similarly, aflibercept was evaluated in the COPERNICUS and GALILEO studies [12, 29]. Since monthly treatment is not feasible in clinical practice, most of the ophthalmologists have been using PRN treatment after a few, usually 3, monthly injections. Treat-and-extend regimen (TER) was described and studied first in AMD treatment in an attempt to personalize the treatment interval and prevent edema-recidivism[30]. In this regimen, the patients receive injections at every visit, but the visit intervals are progressively prolonged to the longest edema-free interval. TER in recent years has gained popularity in RVO-ME treatment [31, 32].

Early development of the epiretinal membrane (ERM) has been associated with BVZ injections in eyes with RVO, whereas a causative relationship has not been established [33]. Researchers have reported, however, the functional and morphological efficacy of combined pars-plana vitrectomy (PPV) and peeling of the internal limiting membrane in CRVO-ME [34–37]. An ERM may result secondary to RVO [38]. The influence of the ERM, however, on the efficacy of treatment of CRVO-ME with anti-VGEF is not well understood.

Here, we conducted a study aimed to describe real-life results of BVZ treatment for CRVO-ME in patients treated according to PRN and TER, as well as to study the possible influence of ERM on BVZ treatment efficacy in a cohort of patients with new-onset CRVO-ME from Southeastern Sweden.

## 2. Materials and Methods

### 2.1. Design and Participants

The present study was a retrospective cohort study, conducted in real-life (IRL) settings at a single center, named the Department of Ophthalmology of the University of Linköping, County of Östergötland, Sweden. Medical records for patients diagnosed with CRVO between February 2012 and March 2018 were reviewed retrospectively. Included in the study were treatment-naïve patients aged 18 years or older with newly diagnosed CRVO with macular edema, treated with only intravitreal BVZ, with the start of treatment within 3 months of the debut of symptoms, and having been followed up for at least 12 months. Patients receiving other anti-VEGF medications, or patients that switched between different treatments, were not included in the study in order to minimize confounding factors. Exclusion criteria were also previous treatment with anti-VEGF, corticosteroids (intravitreally or subtenon), laser treatment in the macular area, or previous vitrectomy. Furthermore, the presence of neovascularization, as well as ME due to other reasons than CRVO (e.g., uveitis, AMD, CSCR, DME, and macular traction), resulted in exclusion from the study. This study was conducted after receiving approval from the Swedish Ethical Review Authority (application no. 2020-03212). All authors have followed the ethical aspects of the study in accordance with the tenets of the Declaration of Helsinki.

### 2.2. Data Collection

Medical data at the time of diagnosis and at 12 months after initiation of treatment were extracted from patient records. The data included age, sex, ophthalmological comorbidities (glaucoma, ERM, neovascularization), best-corrected visual acuity (BCVA) letter score by the Early Treatment Diabetic Retinopathy Study (ETDRS) protocol, central retinal thickness (CRT), and intraocular pressure (IOP). Data concerning treatment details included number of injections, selected treatment regimen, time between the debut of symptoms and treatment start, number of visits, and in some cases, pan-retinal photocoagulation (PRP) or cyclophotocoagulation (cyclodiode laser).

### 2.3. Treatments

Diagnostic measures and treatment were planned and carried out for all cases according to Swedish National Guidelines at the time of study (https://swedeye.org/wp-content/uploads/2016/02/SOTA-retinala-venocklusioner-februari-2016-1.pdf). Indication for treatment in this study was a clinical diagnosis of CRVO established by fundoscopy and/or fundus photography and a decrease in BCVA in the presence of ME as observed using Optical Coherence Tomography (OCT; Spectralis, Heidelberg Engineering, Heidelberg, Germany). There was no lower or upper limit of BCVA defined to initiate treatment. Administration of intravitreal injections of BVZ (1.25 mg in 0.05 ml) began with 3 consecutive injections every 4 weeks. At the first visit, the treatment plan was done and the patient received the first injection. At 4 and 8 weeks, the second and third injections were given without performing any examinations, and these injection-only appointments are not counted in the total number of visits. Four weeks following the third injection, the patient met with an ophthalmologist to evaluate the treatment effect by measurement of BCVA and ME based on an OCT exam. Thereafter, repeated injections continued every 4 weeks until ME resolved. Follow-up visits included the following examinations: BCVA, IOP (measured with a Goldmann applanation tonometer), slit-lamp examination of the anterior and posterior segments, and OCT. After ME had resolved, treatment was continued according to either *pro re nata* or (PRN) treat-and-extend regimen (TER). The treatment protocols followed at the clinic are In TER, once the macula is edema-free, an injection is given and the follow-up interval is extended to 6 weeks. If the macula remains edema-free after 6 weeks, an injection is given (thus an injection every visit) and the follow-up interval is extended by an additional 2 weeks (up to 26 weeks interval). If ME recurs, an injection is given and the visit interval is reduced by 2 weeks (the minimum interval is 4 weeks). Disease activity is assessed by the ophthalmologist and is based on the presence of sight-threatening intraretinal fluid (IRF) or subretinal fluid (SRF) on qualitative OCT at each follow-up visit. No specific predefinite CRT criteria needed to be met in order to establish disease activity.

In the PRN regimen (i.e., injections as needed), the patient is checked every 4–6 weeks, and treatment is given only if the edema recurs. Re-treatment in PRN is based on an ophthalmologist's judgement and the criteria for retreatment in PRN are the presence of new or increased retinal edema (IRF and/or SRF) on qualitative OCT, increased central retinal thickness (CRT), and BCVA decrease considered related to ME. Thus, no predefinite quantitative CRT or BCVA retreatment criteria are indicated.

The assignment to a treatment group was based on intention-to-treat, that is, on the medical decision and plan for treatment established at the first visit. Nevertheless, the real-life settings of the study imply that deviations from the established treatment protocol, in particular regarding the frequency of visits, were tolerated. In the case of the development of neovascularization at the angle or on the iris, a complete PRP was performed. Eyes that developed neovascular glaucoma were treated with cyclophotocoagulation if PRP was contraindicated or not possible to perform.

### 2.4. Statistical Analysis

For statistical analyses, BCVA values from the ETDRS chart were converted to logarithms of the minimum angle of resolution (LogMAR) [39]. Collected data were analyzed from all included eyes. Four subgroups were defined based on the treatment regimen used (TER or PRN) and based on the presence of an ERM during the first 6 months of treatment (ERM or non-ERM). Differences in BCVA, CRT, number of injections, and visits across ERM and non-ERM subgroups were calculated using the independent *t*-test. Differences in the number of injections and visits between PRN and TER were evaluated using the Mann–Whitney test. A *p* ≤ 0.05 significant level was used. Where data was normally distributed, the paired *t*-test was used to compare outcomes at baseline and 12 months in the same eyes. Where data was not normally distributed, the Wilcoxon signed-rank test was used instead.

## 3. Results

In total, 99 patients (53 females, 101 eyes total) were identified for inclusion in this study, based on the diagnosis. Of these, 43 eyes were excluded due to discontinuation of treatment prior to 12 months or due to a switch to other administered drugs. Results reported here are based on data collected from 58 eyes of 56 patients with newly diagnosed CRVO presenting with ME, treated with intravitreal BVZ for at least 12 months. The mean age of the patients was 71 years (range: 34–93 years) at the time of diagnosis. Thirty-one of the patients were women (55%) and 25 were men (45%). Treatment was started in all eyes within 12 weeks of the debut of symptoms.

### 3.1. Administered Treatment and Subgroup Analyses

Thirty-two of the 58 treated eyes (55%) received their first BVZ injection within 4 weeks of the debut of clinical symptoms. The mean number of BVZ injections was 6.3 (range 3–12) at 12 months. Fifteen patients (26%) received ≥9 injections, and 33 patients (57%) received ≤6 injections. The mean number of visits was 7.6 (range 4–11) at 12 months (injections number 2 and 3 are given in injection-only appointments and are not counted in the number of visits).

### 3.2. Regimen Subgroup

**Figure 1:**
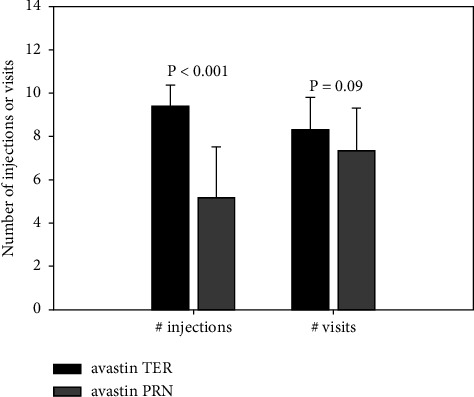
Number of injections and visits according to treatment regimen at 12 months. PRN, *pro re nata* regimen; TER, treat-and-extend regimen. Error bars represent standard deviation.

Forty-two patients were treated according to PRN, and 16 patients according to TER. At 12 months, 5.2 ± 2.3 (mean ± SD) injections were given in the PRN group and 9.3 ± 1.1 in TER (*p* < 0.001). Over the 12 months, a mean of 7.3 and 8.2 visits were made by PRN and TER groups, respectively (*p*=0.09, Figure 1).

### 3.3. ERM Subgroup

Sixteen eyes were included in the ERM group and the remaining 42 were in the non-ERM group. In the ERM subgroup, four of 16 eyes (25%) were treated according to TER, while in the non-ERM subgroup, 12 of 42 eyes (29%) were treated according to TER.

**Figure 2:**
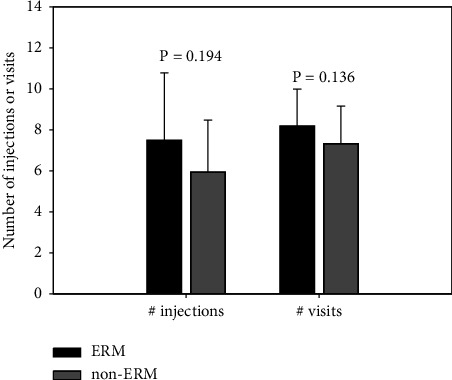
Number of injections and visits according to early presence of epiretinal membrane (ERM) at 12 months. ERM, epiretinal membrane; non-ERM, eyes that did not develop an ERM during the first 6 months of treatment. Error bars represent standard deviation.

At 12 months, 7.2 ± 3.2 (mean ± SD) injections were given in the ERM group and 6.0 ± 2.5 in non-ERM (*p*=0.194). During the 12 months, a mean of 8.2 and 7.3 visits were performed in the ERM and non-ERM groups, respectively (*p*=0.136, Figure 2).

### 3.4. Visual Outcomes and Subgroup Analyses

**Figure 3:**
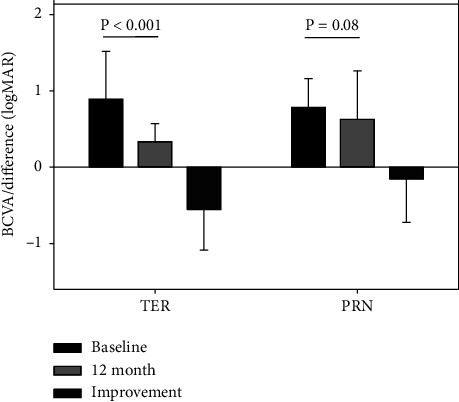
The effect of treatment regimen on BCVA improvement. PRN, pro re nata regimen; TER, treat-and-extend regimen. Error bars represent standard deviation.

**Figure 4:**
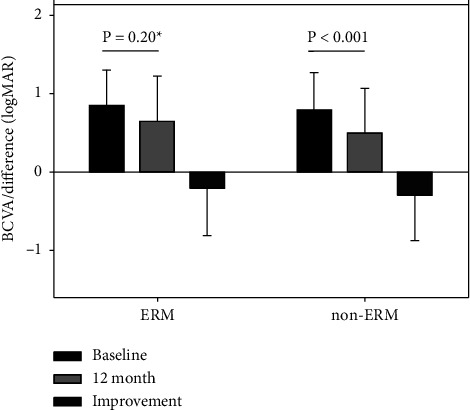
The effect of epiretinal membrane (ERM) presence on BCVA improvement. Change in BCVA in the ERM subgroup was not significant (under-powered due to small group size, 16 subjects, power = 0.13). ERM, epiretinal membrane; non-ERM, eyes that did not develop an ERM during the first 6 months of treatment. Error bars represent standard deviation.

Overall, BCVA improved from 0.81 LogMAR (range 2.3–0.02, SD: ± 0.45) at baseline to 0.54 LogMAR (range 2.3–[−0.14], SD: ± 0.56) at month 12, representing a mean of 13.6 letters gain (*p* < 0.001, Table 1). The mean BCVA at baseline did not differ between the PRN and TER groups. At 12 months, no significant BCVA improvement was noted in eyes treated with PRN (*p*=0.08, Table 1) while for TER, BCVA improved by a mean of 24.6 letters (*p* < 0.001, Table 1, Figure 3). Mean BCVA at baseline did not differ in the ERM and non-ERM groups. At 12 months, the mean change in BCVA was 10.8 letters in the ERM group (*p*=0.197, Table 1). However, the test was underpowered due to the small group size. For non-ERM, BCVA improved by a mean of 14.7 letters (*p* < 0.001, Table 1, Figure 4).

### 3.5. Anatomical Outcomes and Subgroup Analyses

**Figure 5:**
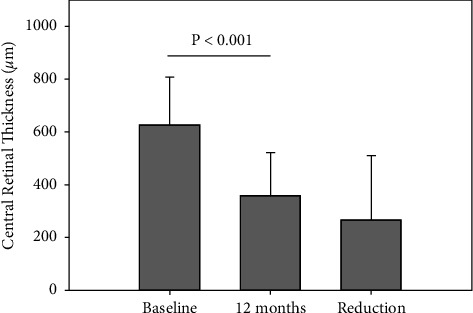
Mean central retinal thickness (CRT) at baseline, after 12 months and the reduction in CRT (12 months minus baseline value). Error bars represent standard deviation.

In all eyes, mean CRT was reduced at 12 months by a mean of 267 *μ*m relative to baseline (*p* < 0.001, Table 2, Figure 5). Mean CRT at baseline did not differ in the PRN and TER groups. At 12 months, mean CRT improved in eyes treated with PRN by a mean of 258 *μ*m (*p* < 0.001, Table 2). For TER, CRT improved by a mean of 288 *μ*m (*p* < 0.001, Table 2). Mean CRT at baseline did not differ in ERM and non-ERM groups. At 12 months, CRT improved in the ERM group by a mean of 255 *μ*m (*p*=0.003, Table 2). In the non-ERM group, CRT improved by a mean of 271 *μ*m (*p* < 0.001, Table 2).

### 3.6. Adverse Events and Associated Procedures

During the 12-month treatment period, no retinal detachment, endophthalmitis, or nonocular side effects were observed. The reported side-effects were eye pain, subconjunctival bleeding, and increased IOP.

### 3.7. Glaucoma

None of the 58 treated eyes had neovascularization at diagnosis. Of 58 eyes, 14 (24%) presented initially with glaucoma or ocular hypertension, which was treated with hypotensive eye drops. Two of these eyes (2 patients) developed neovascular (NV) glaucoma within one year of treatment. Both patients were treated with cyclophotocoagulation. At 12 months, the total number of eyes with glaucoma was 17; three new eyes with glaucoma had developed the neovascular type. They were all treated with PRP. All eyes that developed neovascular glaucoma were in the PRN group; thus, 12% of the eyes treated according to PRN showed neovascular glaucoma at 12 months.

### 3.8. IOP Change

At 12 months, the mean IOP change from baseline in all 58 eyes, including those with NV glaucoma, was +1.98 mmHg (range: −12 to +31, SD ± 7.4). Excluding the five eyes with NV glaucoma at 12 months, the mean IOP change was +0.5 mmHg (range −12 to +20, SD ± 4.6). Six of these 53 eyes (11%) had a decrease in IOP of ≥5 mmHg with a mean of 7.5 injections, and five of 53 eyes (9%) had an increase in IOP of ≥5 mmHg with a mean of 5.0 injections. Nine out of 58 eyes had developed an IOP increase of ≥5 mmHg. In four of these, the cause was thus neovascularization. Of the remaining five eyes, two had glaucoma at diagnosis and additional antiglaucoma drops were needed, while three eyes did not require antiglaucoma treatment.

### 3.9. Neovascularization

At 12 months, a total of seven eyes (7 patients) had developed neovascularization (12% of the total eyes). Five of these eyes had NV glaucoma. Of the 7 eyes, two were treated with cyclophotocoagulation and the remaining 5 eyes with PRP, each receiving one or more laser sessions during the 12 months. In addition to the laser, all eyes with neovascularization were treated with intravitreal bevacizumab as well. All 7 eyes which developed neovascularization during the last 12 months had been undergoing treatment according to the PRN regimen, representing 17% of the eyes in the PRN group, and receiving a mean of 3.7 (range 3–5) injections and 7 (range 4–10) visits during the year. Five of these 7 patients had BCVA ≥38 letters ETDRS at baseline and all 7 had visual deterioration, with BCVA ≤23 letters at 12 months.

## 4. Discussion

Intravitreal anti-VEGF treatment of ME secondary to CRVO has been reported in several studies. Lip et al. reported 1-year outcomes of treatment of ME due to new and chronic CRVO with PRN BVZ in a UK study [40]. CRVO was considered chronic if the duration between diagnosis and treatment start was ≥1 year and if the patient had previously been treated with laser or steroid injections [40]. In our study, CRVO-ME patients who began BVZ treatment within 12 weeks of diagnosis according to either the PRN or TER regimen achieved generally better visual acuity outcomes than reported by Lip et al. [40], where treatment started within 12 months of diagnosis. Median BCVA improved in the present study from 0.72 LogMAR at baseline to 0.37 at 1 year (*p* < 0.001), whereas Lip et al. reported no change in BCVA (median 0.78 LogMAR at both baselines and at 1 year, *p*=0.17). In the present study, a gain of ≥15 and ≥5 letters occurred in 41% and 69% of subjects, respectively, whereas Lip et al. reported 30% and 40%, respectively. In both studies, the anatomical outcome was similar, with median CRT improving from 627 *μ*m at baseline to 359 *μ*m at 1 year in the present study, and from 449 *μ*m to 278 *μ*m as reported by Lip et al. (*p* < 0.001 for both). Whereas Lip et al. treated with a mean of 4.2 injections, we treated with a mean of 6.3 injections (across both regimens). The comparison thus suggests that early treatment and more frequent injections may yield better visual outcomes, despite similar anatomic results. Probably, this reflects the fact that longer-standing macular edema as well as repeated recurrences of macular edema may jeopardize photoreceptor integrity and functions.

A report from Rahimy et al. showed that TER with BVZ and/or ranibizumab was effective in improving visual and morphological outcomes at 1 year in eyes with ME secondary to CRVO [41]. Another study reported that TER stabilized vision with a tendency towards longer treatment intervals over time in patients with CRVO-ME [42]. Although TER was originally designed for neovascular age-related macular degeneration (nAMD) [30], TER with BVZ or ranibizumab over 12 months has been shown as a suitable regimen in the treatment of diabetic macular edema (DME) and ME due to CRVO [41,43]. In our study, only TER gave significant vision improvement in ME secondary to CRVO. This result is in accordance with other studies. In a retrospective study comparing PRN and TER for RVO-ME using intravitreal ranibizumab for at least 1 year, the gain in vision with TER was greater than the gain with PRN, but the difference was not significant. However, the number of patients with CRVO was lower than in the present study [44]. Garcia-Arumi et al. [45], O'Day et al. [32], and Casselholm de Salles et al. [46] also showed that aflibercept given by TER was effective for CRVO-ME.

Several randomized clinical trials (RCTs) have reported a mean improvement in BCVA ranging from +13.9 letters [11] to +16 [47] and +16.2 [48]. Real-life studies usually do not achieve as good results as RCTs; however, our results with TER assessed retrospectively were comparable to the above RCTs, which all used a PRN regimen. Eleftheriadou et al. evaluated the efficacy of aflibercept injections in CRVO-ME according to the TER regimen and similarly reported visual outcomes approaching those of RCTs that used a PRN regimen [31].

Anatomic results in our study show significant improvements in all patients. However, the range of CRT values at 12 months (Table 2) shows that ME has not been adequately treated in at least some of the patients. We believe that this fact is mostly due to the insufficient frequency of injections, which is more evident in the PRN group, and not due to the drug of choice that may only have had a minor influence. The SCORE2-study showed noninferiority of bevacizumab vs aflibercept in CRVO-ME [24]. Lofty et al. show that both bevacizumab and aflibercept are equally effective in RVO-ME, but that bevacizumab needs 1,7 more injections per year to yield the same results [49]. Similarly, Casselholm de Salles et al. concluded that ranibizumab achieved the same results as aflibercept in CRVO-ME given according to TER but with more injections needed [46].

The main problem related to PRN lies in its nature of the reactive treatment, that is the macula experiences repeated recurrences of edema that damage photoreceptors. This might explain our finding that a significant improvement in CRT was not accompanied by a significant increase in BCVA in the PRN group. Our results show clearly that PRN in IRL settings has other limitations due to the difficulty of achieving a sufficient frequency of controls as planned. Only a mean of 7.3 visits were performed in our PRN group, despite a planned visit every 4–6 weeks, which would give an interval of visits between 8 and 13 (as in RCTs). Many studies show that PRN IRL implies a high risk for undertreatment. Further indications of undertreatment are the fact that in our study all eyes that developed neovascularization were treated according to the PRN regimen.

Nonischemic CRVO may develop into ischemic CRVO in about 30% of eyes [50–53], usually with a more rapid visual deterioration. Intraocular anti-VEGF therapy causes the iris neovascularization to regress and the obstruction in the angle to decrease. Bevacizumab has also been suggested as adjuvant therapy for NV glaucoma [54,55]. Our results show that 17% of all eyes in the PRN group developed neovascularization. On the other hand, no case of NV complications was reported in the TER group. These observations suggest that, when treating CRVO-ME with BVZ, TER may be advantageous in preventing neovascularization. Likewise, Garcia-Arumi et al. suggested that TER might be preferred due to a low rate of ocular adverse effects [45].

In a report examining the effects of intravitreal anti-VEGF on IOP change in numerous studies, conflicting results about sustained IOP change were found in 7 studies, concluding that 4–15% of eyes developed a long-term increase in IOP after anti-VEGF injections over 9–24 months. Six other studies, however, did not find any change in IOP after 1–36 months of injections [56]. For example, the IRIS study showed a mean decrease in the intraocular pressure of 0.9 mmHg in eyes treated with 3 types of anti-VEGF injections over at least 12 months in patients with nAMD [57]. A correlation between the number of anti-VEGF injections and long-term IOP increase has been reported in eyes with nAMD and DME treated according to TER followed for more than one year [58]. A sustained rise in IOP of ≥5 mmHg was found in about 5.9% of the cases [58]. In our study, when NV glaucoma as a confounding factor in IOP change was excluded, the mean long-term IOP change at 1 year was a small increase of 0.5 mmHg. Subgroup analysis did not show any correlation between the number of injections and IOP changes.

The possible effect of ERM on the efficacy of anti-VEGF injections in treating macular edema and improving vision is a matter of debate. The influence of ERM on the efficacy of anti-VEGF treatment has previously been reported in AMD and BRVO patients, where no anatomical improvement was observed [59,60]. Here, we found that anatomical outcomes were similar in both the ERM and non-ERM subgroups, but only the non-ERM subgroup had a significant gain in vision at 12 months. The ERM subgroup, however, was too small to evaluate possible vision improvements. No patients had developed ERM with a macular traction at a degree that was judged to affect vision and imply indication for surgery. Non-ERM and ERM subgroups had an almost equal percentage of eyes treated according to the same regimen. The treatment regimen was thus not likely to be a confounding factor that could affect the analysis of ERM subgroup outcomes. Our results show that there was no difference in the quantity of visits between the ERM and non-ERM subgroups. One study that observed the efficacy of pars plana vitrectomy (PPV) with the internal limiting membrane (ILM) peeling in eyes with CRVO-ME and hemi-retinal vein occlusion (HRVO) reported a persistent anatomic improvement over 5 years and a significant improvement in visual acuity in eyes with perfused CRVO and HRVO [37]. In another study, it was suggested that ILM peeling reduces the barrier against the diffusion of fluids toward the vitreous [61]. Others concluded that PPV with peeling of ILM increases retinal oxygenation through the vitreous and thus improves retinal blood flow [62–64]. We hypothesized that the ERM might influence treatment by playing a barrier-like role that could hinder the access of BVZ from the vitreous body to the retinal tissue in the macular area. Our outcomes show, however, that no significant difference regarding the number of BVZ intravitreal injections given could be observed at 12 months, when comparing eyes that developed early ERM with those that did not. Future studies should investigate functional outcomes in a larger population with ERM.

A strength of this study was that the health service in Sweden provides an ideal environment to record IRL results, with conditions free of biases such as affordability and insurance. All patients living in the County of Östergötland were at the time of the study treated at our department, providing a representative study population from a whole region. In addition, our department follows comprehensive and uniform criteria in therapy choice and retreatment decisions. The retrospective design and the IRL settings are usually considered limitations; they may, however, be seen as strengths since they represent important complements to RCTs, confirming the validity of results from RCTs in unselected populations and real-world conditions, raising new questions useful for designing new RCTs, or guiding clinicians in areas where RCTs are lacking. The limitations of the present study included a limited number of eyes and the fact that not all patients followed a strictly planned visit schedule due to the IRL settings of the study. We observed a well-known pattern in clinical practice: the frequency of visits performed is clearly less than the planned frequency. This implies a risk of undertreatment and poor visual outcomes of the treatment. Testing for significant changes in visual acuity in the ERM subgroup was underpowered due to small group size. Also, the cohort analysis was based on an unequal number of eyes in different subgroups. Furthermore, the exclusion of patients that switched to another pharmacological treatment due to insufficient response, done in order to minimize confounding factors, might have influenced the results. Since we report results at 12 months, future prospective studies may be necessary to determine the long-term effect of the ERM on anti-VEGF efficacy.

In conclusion, the results of this study suggest that early treatment with intravitreal bevacizumab, given with an adequate frequency of injections, according to either PRN or TER, resolved edema and stabilized vision in the first 12 months, with TER yielding significant visual improvement and no observed cases of neovascular complications. PRN in IRL may expose the patients to the risk of undertreatment with possible consequent inferior BCVA and a larger risk of neovascular complications. In our study, ERM had no influence on intravitreal bevacizumab efficacy for ME in CRVO during the first year of treatment.

## Figures and Tables

**Table 1:** The effect of ERM presence and regimen of treatment on BCVA improvement.

	Number of eyes	Letters at baseline mean ± SD (range) {LogMAR}	Letters at 12 months mean ± SD (range) {LogMAR}	Letters gained at 12 months mean ± SD (range)	*p* value 0–12 m	≥15 letters gained	≥5 letters gained
**Total**	**58**	**45.2** **±** **20.4 (0-84) {0.81}**	**57.8** **±** **25.1 (0-92) {0.54}**	**13.6** **±** **25 (−40 to +74)**	**<0.001**	**24 (41%)**	**40 (69%)**

PRN	42	45.8 ± 18.7 (6–48) {0.78}	55.2 ± 27.9 (0–92) {0.63}	9.4 ± 25.4 (**−**40 to +74)	0.08	16 (38%)	27 (64%)

TER	16	43.6 ± 24.2 (0–76) {0.89}	68.2 ± 11.3 (48–86) {0.33}	24.6 ± 20.1 (+1 to +65)	<0.001	8 (50%)	13 (81%)

*p* value baseline		0.53					

ERM	16	42.3 ± 21.5 (6–78) {0.85}	53.1 ± 26.6 (3–85) {0.65}	10.8 ± 28.3 (**−**40 to +74)	0.2	6 (38%)	11 (69%)

Non-ERM	42	46.3 ± 19.8 (0–84) {0.79}	61.0 ± 24.2 (0–92) {0.51}	14.7 ± 23.5 (**−**40 to +65)	<0.001	18 (43%)	29 (69%)

*p* value baseline		0.53					

*p* value baseline: significance of the difference in the visual acuity between different subgroups at baseline. *p* value 0–12 m: significance of the difference between baseline and 12 months in the same subgroup. PRN, *pro re nata* regimen; TER, treat-and-extend regimen. ERM, epiretinal membrane; non-ERM, eyes which did not develop an ERM during the first 6 months of treatment.

**Table 2:** Difference in the central retinal thickness (CRT) change between subgroups.

	Number of eyes	Mean CRT at baseline *μ*m (range)	Mean CRT at 12 months *μ*m (range)	Mean reduction CRT, *μ*m (range)	*p* value 0–12 m	Patients with worsening or no reduction CRT (%)
**Total**	**58**	**627 (244, 991)**	**359 (186, 983)**	**267 (−411, +741)**	**<0.001**	**9 (16%)**

PRN	42	631 ± 193 (244, 991)	371 ± 182 (186, 983)	258 ± 258 (**−**411, 741)	<0.001	7 (17%)

TER	16	615 ± 141 (428, 971)	327 ± 84 (237, 537)	288 ± 196 (**−**59, 734)	<0.001	2 (13%)

*p* value baseline		0.74				

ERM	16	639 ± 210 (244, 991)	385 ± 125 (223 to 674)	255 ± 274 (**−**270, 741)	0.003	5 (31%)

Non-ERM	42	622 ± 167 (300, 980)	350 ± 173 (186 to 983)	271 ± 230 (**−**411, 734)	<0.001	4 (10%)

*p* value baseline		0.7				

*p* value, measure the significance of the difference in the CRT change between subgroups at baseline. *p* value 0–12 m = the significance of the difference between baseline and 12 months in the same subgroup. PRN, *pro re nata* regime; TER, treat-and-extend regimen. ERM, epiretinal membrane; non-ERM, eyes which did not develop an ERM during the first 6 months of treatment.

## Data Availability

Data are available on request to PhD Helen Setterud.
